# Cerebellar Integrity in the Amyotrophic Lateral Sclerosis - Frontotemporal Dementia Continuum

**DOI:** 10.1371/journal.pone.0105632

**Published:** 2014-08-21

**Authors:** Rachel H. Tan, Emma Devenney, Carol Dobson-Stone, John B. Kwok, John R. Hodges, Matthew C. Kiernan, Glenda M. Halliday, Michael Hornberger

**Affiliations:** 1 Neuroscience Research Australia, Randwick, New South Wales, Australia; 2 School of Medical Sciences, University of New South Wales, Randwick, New South Wales, Australia; 3 ARC Centre of Excellence in Cognition and its Disorders, Sydney, New South Wales, Australia; 4 Sydney Medical School, University of Sydney, Sydney, New South Wales, Australia; 5 University of Cambridge, Department of Clinical Neurosciences, Cambridge, United Kingdom; University of Ulm, Germany

## Abstract

Amyotrophic lateral sclerosis (ALS) and behavioural variant frontotemporal dementia (bvFTD) are multisystem neurodegenerative disorders that manifest overlapping cognitive, neuropsychiatric and motor features. The cerebellum has long been known to be crucial for intact motor function although emerging evidence over the past decade has attributed cognitive and neuropsychiatric processes to this structure. The current study set out i) to establish the integrity of cerebellar subregions in the amyotrophic lateral sclerosis-behavioural variant frontotemporal dementia spectrum (ALS-bvFTD) and ii) determine whether specific cerebellar atrophy regions are associated with cognitive, neuropsychiatric and motor symptoms in the patients. Seventy-eight patients diagnosed with ALS, ALS-bvFTD, behavioural variant frontotemporal dementia (bvFTD), most without *C9ORF72* gene abnormalities, and healthy controls were investigated. Participants underwent cognitive, neuropsychiatric and functional evaluation as well as structural imaging using voxel-based morphometry (VBM) to examine the grey matter subregions of the cerebellar lobules, vermis and crus. VBM analyses revealed: i) significant grey matter atrophy in the cerebellum across the whole ALS-bvFTD continuum; ii) atrophy predominantly of the superior cerebellum and crus in bvFTD patients, atrophy of the inferior cerebellum and vermis in ALS patients, while ALS-bvFTD patients had both patterns of atrophy. Post-hoc covariance analyses revealed that cognitive and neuropsychiatric symptoms were particularly associated with atrophy of the crus and superior lobule, while motor symptoms were more associated with atrophy of the inferior lobules. Taken together, these findings indicate an important role of the cerebellum in the ALS-bvFTD disease spectrum, with all three clinical phenotypes demonstrating specific patterns of subregional atrophy that associated with different symptomology.

## Introduction

Amyotrophic lateral sclerosis (ALS) and frontotemporal dementia (FTD) are multisystem neurodegenerative disorders that are increasingly recognised to lie on two ends of a disease spectrum, and demonstrate overlapping clinical, pathological and genetic characteristics [1]–[3]. From a clinical perspective, 10–15% of patients with behavioural variant frontotemporal dementia (bvFTD) demonstrate motor impairment that meet criteria for a diagnosis of ALS [4]–[6], but subtle motor system dysfunction is present in a significant proportion of patients who do not reach diagnostic criteria [7]. Similarly, while 15% of patients with ALS manifest cognitive and neuropsychiatric changes characteristic of bvFTD [8], [9], cognitive impairment is also present in a further 35% of ALS patients that do not meet bvFTD criteria [8], [10]. Neuropsychiatric features (disinhibition, apathy, loss of sympathy, stereotypical behaviour, dietary changes and executive deficits) [11] are also observed, particularly in patients who develop ALS-bvFTD [5] and have the *C9ORF72* mutation [12], [13].

Historically, the cognitive and neuropsychiatric features in these syndromes have largely been attributed to involvement of changes in the prefrontal cortex [14], [15], although there is increasing evidence to suggest that degeneration in the cerebellum and subcortical regions also play a role [16], [17]. In particular, the cerebellum, long recognized as crucial for intact motor control and coordination, has become increasingly aligned with cognitive and neuropsychiatric processes, reawakening interest in this region for non-motor functions [18], [19]. Given it has multiple reciprocal connections with diverse cortical brain regions [20], [21], the cerebellum is thought to function as a relay station important for regulating a variety of neural tasks, including higher cognitive and neuropsychiatric processes. Functional neuroimaging and connectivity studies in healthy humans suggest a topographic organization in the cerebellum, with different subregions involved in sensorimotor and cognitive processes - sensorimotor function represented in superior lobules I–V and inferior lobule VIII, and cognitive processing represented in lobules VI and VII [21]–[24]. Despite this knowledge, the relationship between subregional atrophy in the cerebellum and cognitive and neuropsychiatric symptoms in bvFTD and ALS is essentially unexplored.

Interestingly, cerebellar atrophy has been observed in ALS and bvFTD [25], [26], though comparisons across the different ALS-bvFTD syndromes and any associations with specific clinical symptoms have not been explored. As such, the present study set out to examine grey matter integrity in subregions of the cerebellum in three distinct ALS-bvFTD cohorts, to assess any associations with clinical deficits in motor, cognitive and neuropsychiatric function in the patients. Based on previous findings in healthy humans [21], [23], [27], we predicted that all three groups would show atrophy in the cerebellum, with greater involvement of lobules VI and VII in bvFTD, and of the superior lobules I–V and inferior lobule VIII in ALS. Against the growing body of pathological and genetic evidence suggesting that ALS and bvFTD lie on two ends of the same disease spectrum [28], [29], we hypothesized that ALS-bvFTD patients would reveal atrophy across the cerebellar subregions affected in ALS and bvFTD.

## Methods

### Case Selection

A total of 78 participants took part in this study. Patients were recruited in a consecutive fashion from the FTD Research Clinic, FRONTIER, as well as specialist ALS multidisciplinary clinics in Sydney, resulting in a sample of 23 bvFTD, 16 ALS-bvFTD, 23 ALS and 16 controls. All FTD patients met current consensus criteria for bvFTD [11], showing the progressive neuropsychiatric and/or cognitive decline characteristic of this dementia subset, including some to all of the following: disinhibition, apathy, inertia, loss of empathy, perseveration, stereotypy and dysexecutive syndromes. Patients also met criteria of atrophy localised to frontal and/or temporal lobes via MRI. The ALS patient group was classified as definite or probable ALS, according with El Escorial Criteria Revised [30]. These criteria required either clinical or electromyographic evidence of combined upper and lower motor neuron dysfunction, in a region under spinal or bulbar innervation. Respiratory function was above 70%, indicated by forced vital capacity (FVC), with no indication of nocturnal hypoventilation. The ALS-bvFTD group comprised patients who met diagnostic criteria for both syndromes, showing both upper and lower motor neuron signs and progressive neuropsychiatric/cognitive dysfunction. Diagnosis was established by consensus among senior neurologists (MCK, JRH) and neuropsychologist based on clinical investigations, cognitive assessment, carer interviews, and evidence of atrophy on structural neuroimaging. No patients were included in the bvFTD group who manifested any ALS symptomology, and no patients had any additional neurological or motor syndromes. A group of 16 healthy adults were included as controls. Groups were matched for age, gender, education and duration of disease. Testing and scanning was conducted at the first clinic visit of each patient.

### Screening for C9ORF72 repeat expansions

Blood collection for genetic screening was performed after informed consent. Patients were screened using the repeat primed polymerase chain reaction (PCR) procedure described previously [31], which is based on the protocol of Renton and colleagues [32]. A patient's DNA sample was deemed positive for the *C9ORF72* repeat expansion if it contained an allele with >30 repeats. 2 bvFTD and 1 ALSFTD participant had the *C9ORF72* gene abnormality.

### Ethics Statement

Ethics approval was obtained from the Human Research Ethics Committee of South Eastern Sydney/Illawarra Area Health Service (HREC 10/126, 10/092 & 10/022). Research was conducted following the ethos of the Declaration of Helsinki. Written consent, either from patient or family, was obtained for each participant in the study.

### Test Selection

The Addenbrooke's Cognitive Examination Revised (ACE-R), the Cambridge Behavioural Inventory Revised (CBI-R) and the Amyotrophic Lateral Sclerosis Functional Rating Score-Revised (ALSFRS-R) were selected on the basis of their high sensitivity, specificity and feasibility for cognitive, neuropsychiatric and motor symptoms, respectively.

The ACE-R is a test that detects early cognitive impairment with 94% sensitivity and 89% specificity [33] and has been well validated across various neurodegenerative diseases [34]–[37]. The participant works through a battery of items designed to reveal levels of functioning across five subscales: attention & orientation, memory, fluency, language and visuospatial cognition. The total possible score is 100, with higher scores denoting more preserved cognitive abilities. Scores below 88 are indicative of cognitive impairment [33].

The CBI-R is a 45-item carer questionnaire mapping the neuropsychiatric topography of the participant and any material impact on daily life. Each item, a given behaviour, is ascribed a frequency rating (0–4) – 0 indicating no impairment, 1 a rare occurrence (a few instances per month), 2 a repeated occurrence (a few instances per week), 3 a daily occurrence, and 4 a constant occurrence. The CBI-R stands corroborated by the Neuropsychiatric Inventory (NPI) as an effective measure of neuropsychiatric symptoms [38]. The maximum score is 180, signifying absolute behavioural and psychological dysfunction (Results are reported herein as percentages, for simplicity). Thus higher scores in CBI-R indicate greater impairment, in contrast with grading of ACE-R scores.

Motor functional status in ALS and ALS-bvFTD patients was assessed using the Amyotrophic Lateral Sclerosis Functional Rating Score-Revised (ALSFRS-R) [39]. ALSFRS-R items were also grouped into subscores: bulbar, fine motor, gross motor and respiratory, each scored out of 12 points. The maximum ALSFRS-R total was 48 points with a greater reduction in the ALSFRS-R total indicating greater motor disability. ALSFRS data was available for the 23 ALS-bvFTD and ALS patients only.

### Demographics, cognitive, neuropsychiatric and motor analyses

Data were analysed using IBM SPSS 20.0. A priori, variables were plotted and checked for normality of distribution by Kolmogorov-Smirnov tests. Parametric demographic data (age, education), neuropsychological (ACE-R) and neuropsychiatric (CBI-R) data were compared across the four groups (ALS, bvFTD, ALS-bvFTD and controls) via one-way ANOVAs followed by Tukey HSD posthoc tests. Variables revealing non-normal distributions were log transformed and the appropriate log values were used in the analyses, but Table 1 reports their original values to facilitate clinical interpretation. Variables showing non-parametric distribution after log transformation were analysed via Chi-square (gender) and Kruskal-Wallis & Mann-Whitney U (disease duration) tests.

**Table 1:** Demographics, cognition and neuropsychiatric measures in ALS, ALS-bvFTD, bvFTD and control groups.

	ALS (n = 23)	ALS-bvFTD (n = 16)	bvFTD (n = 23)	Controls (n = 16)
**Age (years)**	61±11.5	63±7.3	62±10.1	64±5.1
**Education (years)**	13±3.3	13±3.6	12±3.1	14±1.7
**Gender (M/F)**	14/9 (1.6)	11/5 (2.2)	15/8 (1.9)	8/8 (1)
**Disease duration (years)**	4±4.5	3±2.2	4±2.4	N/A
**Onset (limb:bulbar)**	19∶4	8∶8	N/A	N/A
**ACE-R (total score; 0–100)**	89±10^b, c^	62±19^a, b, d^	74±16^a, c, d^	95±4^b, c^
**CBI-R (total score: 0–180)**	31±20^a, b^	49±22^a, b^	73±34^a, b, c, d^	8.6±11^b, c, d^
**ALSFRS-R (total score; 0–48)**	37±7.7	44±3.3	N/A	N/A

Data are presented as mean ± standard deviation. Differences between groups are represented as ^a^ p<0.05 compared to controls; ^b^ p<0.05 compared to bvFTD; ^c^ p<0.05 compared to ALS-bvFTD; ^d^ p<0.05 compared to ALS. N/A – not applicable.

### Imaging Acquisition

Subjects were scanned using a 3T Philips MRI scanner. T1-weighted acquisition: coronal orientation, matrix 256×256×200, 161 mm^2^ in-plane resolution, slice thickness 1 mm, TE/TI = 2.6/5.8 ms.

### Voxel-based Morphometry (VBM) Analysis

Voxel-based morphometry (VBM) was conducted on the three dimensional T1-weighted scans, using the FSL-VBM toolbox in the FMRIB software library package (http://www.fmrib.ox.ac.uk/fsl/). The first step involved extracting the brain from all scans using the BET algorithm in FSL, using a fractional intensity threshold of 0.22 [40]. Each scan was visually checked after brain extraction, both to ensure that no brain matter was excluded, and no non-brain matter was included (eg. skull, optic nerve, dura mater).

A grey matter template, specific to this study, was then built from canvassing 10 scans from each group (total n = 40). An equal amount of scans across groups was used to ensure equal representation, and thus avoid potential bias toward any single group's topography during registration. Template scans were then registered to the Montreal Neurological Institute Standard space (MNI 152) using non-linear b-spline representation of the registration warp field, resulting in study-specific grey matter template at 2×2×2 mm^3^ resolution in standard space. Simultaneously, brain-extracted scans were also processed with the FMRIB's Automatic Segmentation Tool (FAST v4.0) [41] to achieve tissue segmentation into CSF, grey matter and white matter. Specifically this was done via a hidden Markov random field model and an associated Expectation-Maximization algorithm. The FAST algorithm also corrected for spatial intensity variations such as bias field or radio-frequency inhomogeneities in the scans, resulting in partial volume maps of the scans. The following step saw grey matter partial volume maps then non-linearly registered to the study-specific template via non-lia b-spline representation of the registration warp. These maps were then modulated by dividing by the Jacobian of the warp field, to correct for any contraction/enlargement caused by the non-linear component of the transformation [42]. After normalisation and modulation, smoothing the grey matter maps occurred using an isotropic Gaussian kernel (standard deviation  = 3 mm; full width half maximum = 8 mm).

Statistical analysis was performed with a voxelwise general linear model. Significant clusters were formed by employing the threshold-free cluster enhancement (TFCE) method [43]. The TFCE method is a cluster-based thresholding method which does not require the setting of an arbitrary cluster forming threshold (e.g. t,z). Instead, it takes a raw statistics image and produces an output image in which the voxel-wise values represent the amount of cluster-like local spatial support. The TFCE image is then turned into voxel-wise p-values via permutation testing. We employed a permutation-based non-parametric testing with 5000 permutations [44]. All group comparisons included age as a covariate.

All patient-control group comparisons were tested for significance at p<0.05, corrected for multiple comparisons via Family-wise Error (FWE) correction across space. The inter-patient comparison did not survive FWE correction and were tested at a significance level of p<0.01, false discovery rate (FDR) corrected, and a cluster threshold of 20 contiguous voxels.

In a next step, correlations between performance on clinical scores and cerebellar regions of grey matter atrophy were investigated across all groups. For statistical power, a covariate only statistical model with a (1) t-contrast was used, providing an index of association between grey matter intensity and clinical scores. The clinical scores were included separately in the model, and age was included as a covariate in all analyses given the large range for the participants.

### Region-of-Interest Mask

**Figure 1:**
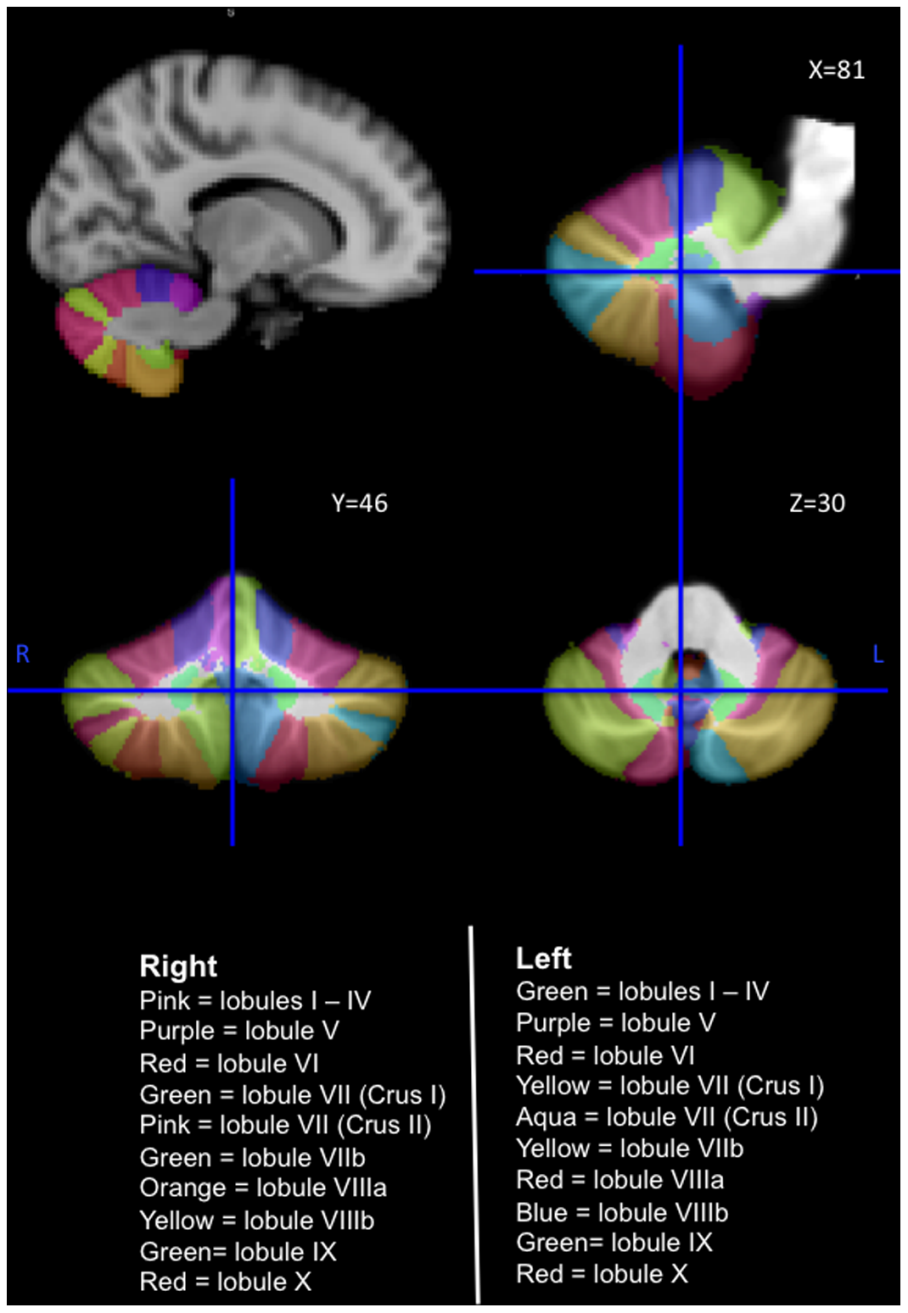
The human cerebellum with lobules I–X labelled from the probabilistic atlas template (SUIT) of the human cerebellum (http://www.icn.ucl.ac.uk/motorcontrol/imaging/propatlas.htm). **L** Left Hemisphere; **R** Right Hemisphere.

A region-of-interest (ROI) mask was created for subregions of the cerebellum (Figure 1). The lobules mask includes lobules I–X; the vermis mask included vermis VI–X; the crus mask included crus I–II. The cerebellar subregions were established using a validated probabilistic atlas of the human cerebellum (http://www.icn.ucl.ac.uk/motorcontrol/imaging/propatlas.htm).

## Results

### Demographics, cognitive, neuropsychiatric and motor profiles

Although there were no significant differences in demographic variables across patients and controls (Table 1), patient groups differed significantly on their general cognitive measures (ACE-R; p<.001). Post-hoc comparisons established that both FTD groups had significant deficits compared to controls (p<0.001), while ALS patients performed similar to controls (p>0.1) and higher than bvFTD and ALS-bvFTD patients (p<.005). ALS-bvFTD patients performed worse than remained bvFTD (p = 0.005).

At a neuropsychiatric level, the groups differed significantly on the CBI-R (p<0.001). Post-hoc comparisons showed controls had a lower score compared to all patients groups (p<0.05); bvFTD patients had higher scores than ALS-bvFTD (p<0.05) and ALS (p<0.001), and no significant difference was found between ALS-bvFTD and ALS scores (p = >0.1). These results confirm that bvFTD patients manifested the worst neuropsychiatric disturbances, with ALS and ALS-bvFTD showing milder neuropsychiatric changes compared to controls.

Motor functional status measured with the ALSFRS-R demonstrated no significant difference between ALS-bvFTD and ALS (p>0.01).

### Voxel-Based Morphometry

To simplify the recognition of anatomical subregions involved in relation to the mask for lobule VII (Crus) placed on the images, the present study refers to the cerebellar subregions as the ‘Superior’ (lobules I–VI), the ‘Crus’ (lobules VIIA) and the ‘Inferior’ (lobules VIIB–X).

#### Subregional cerebellar atrophy in ALS, ALS-bvFTD and bvFTD (Table 2, Figure 2)

**Figure 2:**
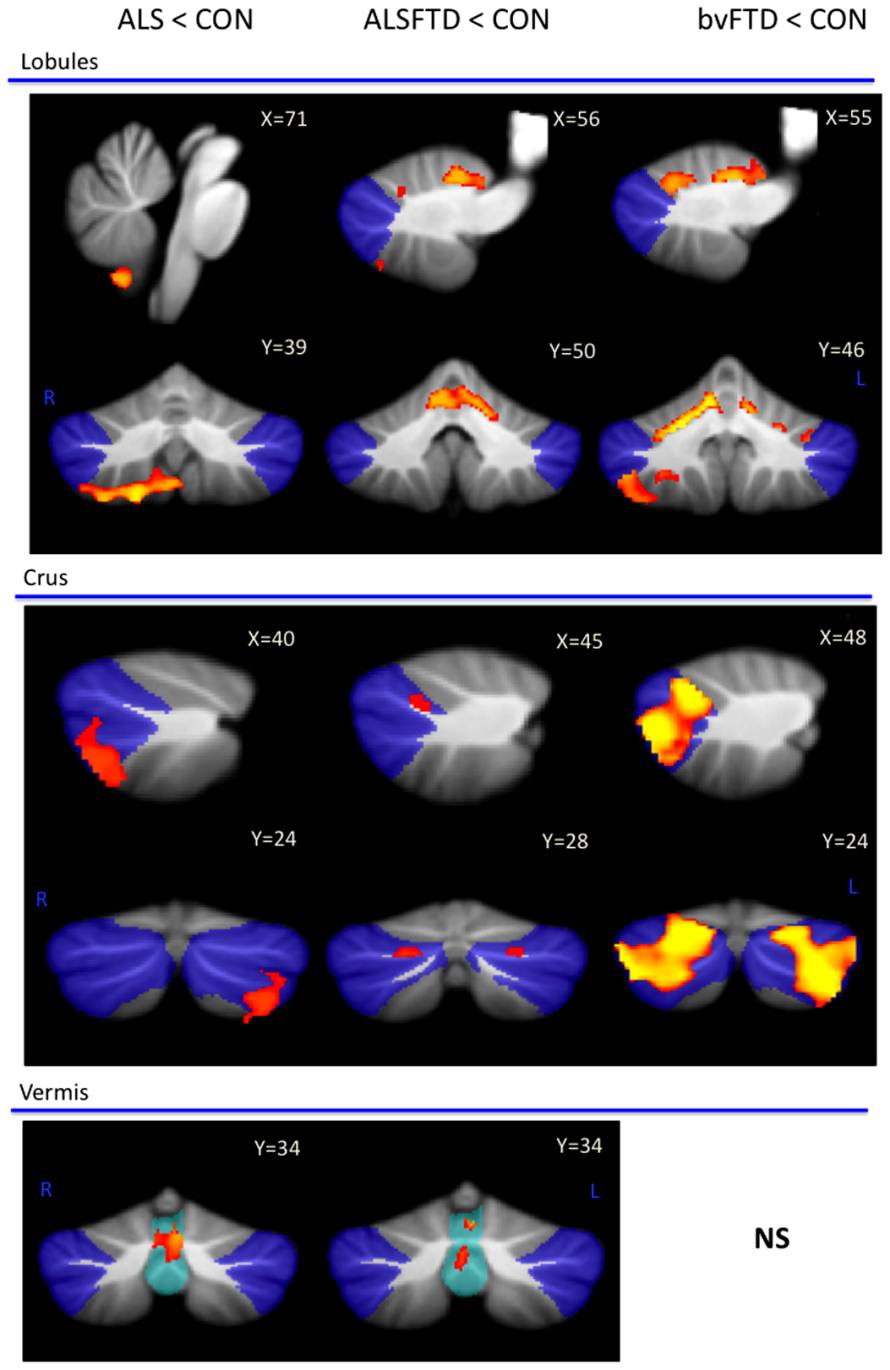
Voxel-based morphometry (VBM) findings for ALS, ALS-bvFTD and bvFTD in comparison to controls. VBM findings are shown in red-yellow; coloured voxels show regions that were significant in the analysis at p<0.05 family-wise error (FEW) corrected. All clusters reported t>3.50. Clusters are overlaid on the MNI standard brain with a mask for lobule VII (crus 1, 2 and VIIb) shown in blue and a mask for the vermis shown in light blue. - No significance; **L** Left Hemisphere; **R** Right Hemisphere.

**Table 2:** Voxel-based morphometry (VBM) atrophy of cerebellum subregions in ALS, ALS-bvFTD and FTD in comparison to controls.

Cerebellar subregions	ALS	ALS-bvFTD	bvFTD
I–IV *	-	Bilateral (medial)	Bilateral (medial)
V *	-	Bilateral (medial)	Bilateral (medial)
VI *	-	Left and very mild right (medial)	Bilateral (medial)
**VII (Crus 1)**	-	Bilateral (medial)	Bilateral (both)
**VII (Crus 2)**	Left Crus 2 (lateral)	Mild bilateral (medial)	Bilateral (both)
**VIIB**	Bilateral (lateral)	Mild left (medial)	Bilateral (both)
VIIIA *	Right (lateral)	Left (lateral)	Right (both)
VIIIB *	Right (lateral)	-	Right (medial)
**IX**	Right (lateral)	-	-
**X (Flocculonodular)**	-	-	-
**Vermis**	VI, VIIb, Crus 2	VI, VIIb, VIIIa	-
**MNI Coordinates X, Y, X**			
**Lobules**	71,30,19	56,50,56	55,46,40
**Crus**	40,24,27	46,28,42	48,24,40
**Vermis**	65,31,48	67,34,42	N/S

All results FEW corrected at p<0.05 and reported at t>3.50.

Cerebellar subregions are grouped based on functional neuroimaging and connectivity studies indicating ‘sensorimotor’ cerebellar regions (marked *) and ‘cognitive’ cerebellar subregions (boxed) [21]–[23]. Within the cerebellar lobules, atrophy of the medial (deeper cerebellar regions), lateral (external cerebellar regions) or both (across medial and lateral) regions are noted. N/S Not significant.

In comparison to controls, ALS patients showed marked grey matter atrophy in the inferior lobules (VII–IX) and vermis, and some changes in the crus. By contrast, bvFTD patients showed significant bilateral grey matter atrophy in the superior lobules (I–VI) and crus with some involvement of the inferior lobule (VII–VIII) and no changes in the vermis. ALS-bvFTD patients showed grey matter atrophy in the superior lobules (I–VI), crus and vermis.

#### Comparison of cerebellar atrophy between patient groups (Table 3, Figure 3)

**Figure 3:**
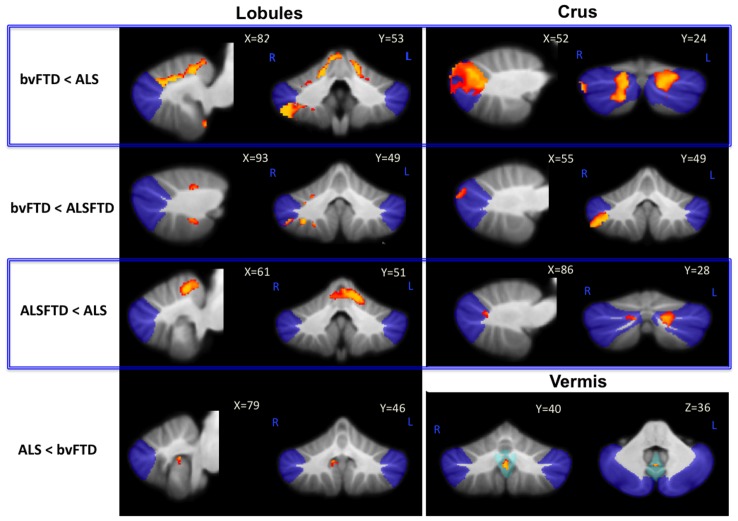
Unique cerebellar grey matter atrophy across patient groups. Voxel-based morphometry findings contrasting grey matter atrophy in the a) lobules; b) crus; c) vermis across groups. Clusters are overlaid on the MNI standard brain with a mask for lobule VII (crus 1, 2 and VIIb) shown in blue and a mask for the vermis shown in light blue. Coloured voxels show regions that were significant in the analysis for p<0.05 family-wise error (FEW) corrected. **NA** Not applicable; - No significance; **L** Left Hemisphere; **R** Right Hemisphere.

**Table 3:** Voxel-based morphometry (VBM) findings contrasting grey matter atrophy in the lobules crus and vermis between patient groups (p<0.05).

	ALS	ALS-bvFTD	bvFTD
**ALS**	N/A	Bilateral I–IV, Left V, Bilateral Crus 1 (mild right), Left Crus 2	Bilateral I–VI, Bilateral Crus 1, Right Crus 2, Right VIIb, VIIIa, VIIIb
**ALS-bvFTD**	-	N/A	Right VI, Right Crus 2, Right VIIb, VIIIa, VIIIb
**bvFTD**	Right lobule IX (mild), Vermis VIIIa, VIIIb, IX	-	N/A

##### ALS vs. bvFTD

Direct comparisons of ALS and bvFTD patients revealed more grey matter atrophy in the right inferior lobule (IX) and vermis in ALS compared to bvFTD. By contrast, bvFTD patients showed substantially more atrophy than ALS in the bilateral superior lobules (I–VI), crus and also the right inferior lobules (VII–VIII).

##### ALS vs ALS-bvFTD

A comparison of ALS and ALS-bvFTD showed greater atrophy in bilateral superior lobules (I–V) and crus in ALS-bvFTD compared to ALS. The reverse contrast revealed no significant differences between ALS and ALS-bvFTD.

##### bvFTD vs ALS-bvFTD

Finally, in comparison to ALS-bvFTD, bvFTD cases had greater right hemisphere atrophy in superior lobule VI, crus and inferior lobules VII–VIII. The reverse contrast revealed no significant differences between ALS-bvFTD and bvFTD.

#### Grey matter volume correlates with measures of cognition, neuropsychiatric and motor function (Table 4, Figure 4)

**Table 4:** Voxel-based morphometry (VBM) findings demonstrating grey matter volumes in cerebellum subregions showing a significant correlation with ACE-R, CBI-R and ALSFRS-R (p<0.05).

Cerebellar subregions	ACE-R	CBI-R	ALSFRS-R
I–IV *	Bilateral (medial)		-
V *	Bilateral (medial)	Mild right (medial)	Right (lateral)
VI *	Bilateral (medial)	Bilateral (medial)	Bilateral (lateral)
**VII (Crus 1)**	Bilateral (both)	Very mild bilateral (medial)	-
**VII (Crus 2)**	Bilateral (both)	-	Left (lateral)
**VIIB**	Mild bilateral (medial)	-	Bilateral (lateral)
VIIIA *	Mild right (medial)	-	Right (lateral)
VIIIB *	Mild right (medial)	-	Right (lateral)
**IX**	Mild right (medial)	-	Right (lateral)
**X (Flocculonodular)**	-	-	-
**Vermis**	-	-	VIIIa, VIIIb, IX

Cerebellar subregions are grouped based on functional neuroimaging and connectivity studies indicating ‘sensorimotor’ cerebellar regions (marked *) and ‘cognitive’ cerebellar regions (boxed). Within cerebellar lobules, atrophy in the medial (deeper cerebellar regions), lateral (external cerebellar regions) or both (across medial and lateral) regions are noted.

**Figure 4:**
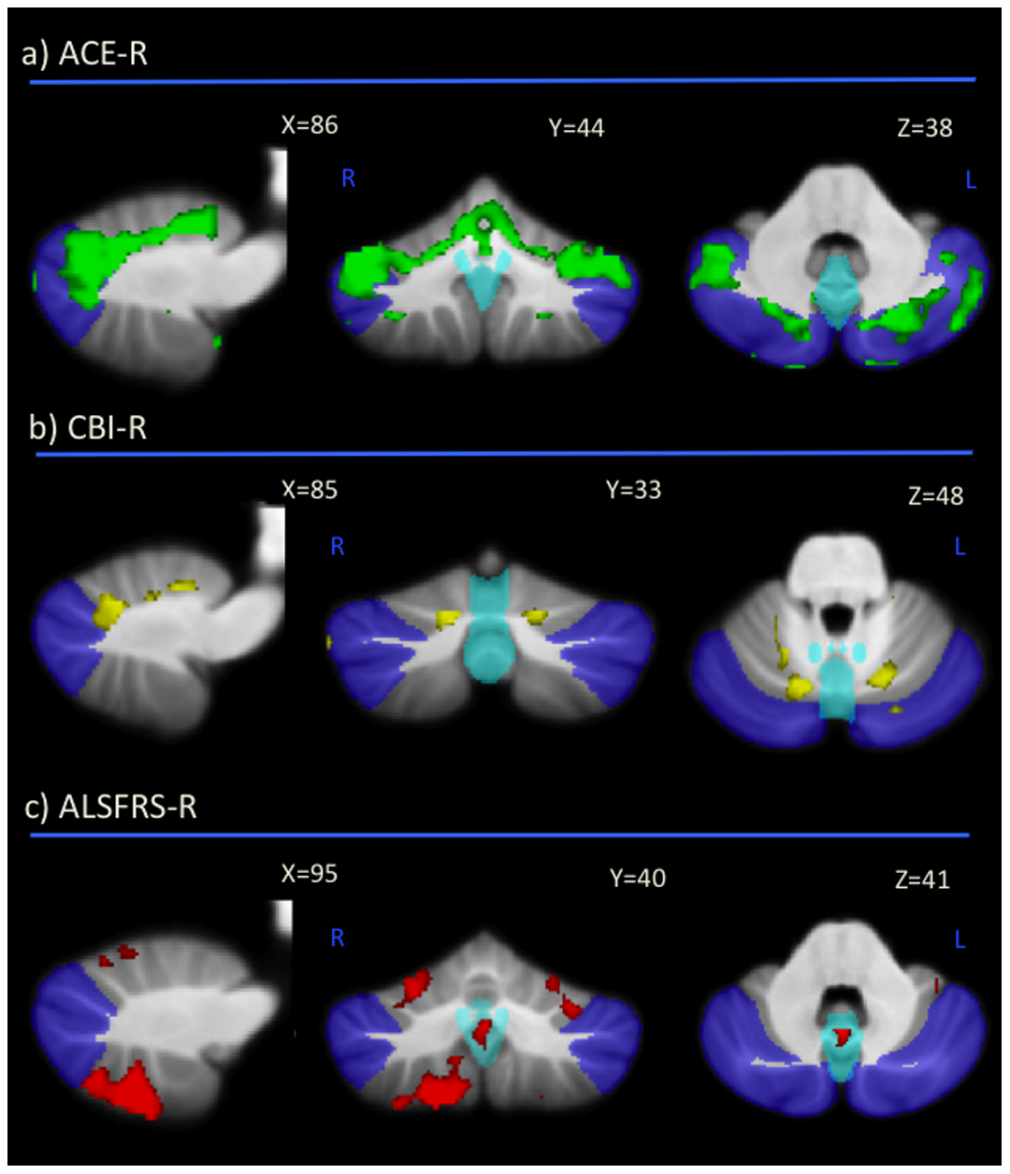
Correlation between cerebellar grey matter volume and a) ACE-R (green); b) CBI-R (light blue); c) ALSFRS-R (red) scores. Clusters are overlaid on the MNI standard brain with a mask for lobule VII (crus 1, 2 and VIIb) shown in blue and a mask for the vermis shown in light blue. Coloured voxels show regions that were significant in the analysis for p<0.05 family-wise error (FEW) corrected. L Left Hemisphere; R Right Hemisphere.

Correlations between cerebellar atrophy and general cognitive (ACE-R) and neuropsychiatric measures (CBI-R) were examined across all groups. Correlations between cerebellar atrophy and motor functional scores (ALSFRS-R) were investigated in a subset of ALS and ALS-bvFTD patients only. ACE-R scores (green colour, Figure 4a) correlated significantly with grey matter volumes in the bilateral superior lobules and crus and to a much lesser degree in the inferior lobules while CBI-R measures (yellow colour, Figure 4b) were associated with grey matter volumes in bilateral, superior lobule VI only. ALSFRS-R scores (red colour, Figure 4c) demonstrated a significant correlation with grey matter volumes in the inferior lobules and vermis and to a lesser degree in the superior lobules.

## Discussion

The present series of investigations undertaken in a large cohort of ALS and FTD patient cohorts has established: i) significant cerebellar atrophy in all ALS-bvFTD syndromes; ii) differences between syndromes in the cerebellar subregions affected, with bvFTD patients showing widespread atrophy predominantly in the superior cerebellum, ALS patients showing atrophy in the inferior cerebellum and vermis, and ALS-bvFTD patients revealing a pattern of atrophy similar to both bvFTD and ALS. Finally, we report that the degeneration of particular cerebellar subregions impacts on cognitive, neuropsychiatric or motor performance in ALS-bvFTD syndromes.

Although subcortical regulatory circuits are required for the normal daily performance of all tasks and functions, subcortical and cerebellar degeneration have been largely ignored in ALS-bvFTD syndromes. Indeed, grey matter structural analyses have focused mostly on cortical regions affected in bvFTD and ALS patients [14], [15], [25], [26], [45]–[52]. Degeneration of the cerebellum in ALS and FTD was reported in two previous studies [25], [26] that showed grey matter atrophy in the right lateral cerebellum in bvFTD patients with moderate to severe cognitive impairment [25], and in ALS patients with no signs of cognitive deficits [26]. An informal retrospective visual inspection of MRI figures revealed that while cerebellar atrophy was also present in many previous studies, this change had not been highlighted [14], [45], [48]–[50], [52]. Investigations into the neural basis of ALS and bvFTD symptoms have focused mainly on grey and white matter changes in cortical brain regions where cognitive and neuropsychiatric changes correlate with frontal and temporal grey matter volumes, particularly on the right [47], [53], [54], while cortical atrophy is not correlated with motor neuron function [26], [55]. Our results show that the cerebellum is clinically relevant by demonstrating, for the first time, that regional atrophy in this structure correlates with cognitive, neuropsychiatric and motor features in ALS and bvFTD. This implicates the cerebellum in cognitive, neuropsychiatric and motor deficits in these disorders.

At a cognitive level, the crus and lobules VI and VIIb are cerebellar subregions that have been shown to be recruited during cognitive tasks involving language, working memory, visual spatial and executive function in healthy humans using functional MRI techniques [22], [23], [27]. Our results show grey matter atrophy in these cerebellar subregions correlated with performance on a broad measure of cognitive function, the ACE-R. The crus forms closed-loop connections with cortical regions not directly involved in sensorimotor processing [20], [21], [56] and damage to this cerebellar subregion gives rise to cognitive impairment without noticeable signs of motor deficits [57]–[59]. Importantly, atrophy of the crus was most pronounced in patients with bvFTD, who also showed the most severe cognitive and neuropsychiatric deficits, whereas the crus and ACE-R test scores were more preserved in ALS-bvFTD and ALS patients. Cerebellar lobules VI and VIIb are also involved in cognitive function but unlike the crus, these lobules also have reciprocal connections with sensorimotor, visual and auditory cortices [20], [21], [56]. We found greater left hemisphere atrophy in lobules VI and VIIb in ALS-bvFTD compared to ALS which, based on functional neuroimaging in healthy humans [19], would suggest greater visuospatial impairment in this cohort. Indeed the visuospatial subscore of the ACE-R (Table S1) confirms this by showing more visuospatial impairment in ALS-bvFTD compared to ALS. Similarly, language impairment has been associated with greater right hemisphere changes in lobules VI and crus [19], [57], [60]. In the present study these subregions were more affected in bvFTD compared to the other patient groups.

Neuropsychiatric disorders are frequently identified in individuals with cerebellar abnormalities but apart from schizophrenia, which is associated with atrophy of the vermis, cerebellar changes in other neuropsychiatric disorders are not well-characterized (see [61], [62]). To date, there has been little focus on this relationship in ALS-bvFTD syndromes. A VBM study investigating loss of insight in FTD showed atrophy of the ventromedial prefrontal cortex but also the cerebellar superior lobules, both of which correlated with the degree of insight in FTD patients [63]. More recently, cerebellar changes relating to neuropsychiatric changes have achieved more prominence in FTD patients with *C9ORF72* mutations. Psychiatric disturbances are more common in patients with *C9ORF72* mutations and cerebellar atrophy is also common in patients with *C9ORF72* mutations [64]–[66]. Still, to date, no direct relationship between the two has been established. More interestingly, a recent study suggested that the cerebellum is more severely affected in sporadic cases than in those with the mutation [67]. While we confirm cerebellar involvement, these findings raise questions regarding cerebellar contributions to neuropsychiatric processes and future studies could further investigate neuropsychiatric correlates with cerebellar subregions in sporadic and *C9ORF72*-mutation cases to further disentangle the relationship between the *C9ORF72* mutation, psychiatric disturbances and the cerebellum.

The inferior cerebellum is routinely recruited during motor tasks [68], [69]. Neurophysiological studies have shown electrical stimulation in this region evokes limb movements [68] and neuroimaging studies have demonstrated grey matter correlates with upper extremity function [69]. We found that atrophy of the inferior cerebellum was associated with motor dysfunction, with marked atrophy in the inferior lobules and vermis in ALS patients. Nevertheless, resting-state functional connectivity and cerebellar lesions in humans have also implicated the superior cerebellum in sensorimotor function [23], [24] due to the overlapping functional connectivity between this subregion with the motor cortices [21]. While the absence of atrophy in the superior cerebellum in the present ALS cohort appears contradictory to these findings, patients with ALS have been found to have no significant atrophy in the motor cortices, with atrophy in these regions associated more with cognitive and neuropsychiatric changes rather than motor impairment [53], [55]. Importantly, the superior cerebellum has been implicated in classic cerebellar motor symptoms such as ataxia [70]. None of our patients had clinical signs of ataxia, which does, however not preclude that ALS-bvFTD continuum patients might have more subtle impairments of motor coordination. Interestingly, bvFTD patients showed the most significant superior cerebellar atrophy of all patient groups, which would suggest that compared to ALS and ALS-bvFTD they should show subtle motor coordination problems. This would be akin to the minor motor cortical deficits in bvFTD that do not meet diagnostic criteria for ALS [7]. Clearly, examination of cerebellar motor features (i.e. ataxia) in detail would be important to follow-up.

On a more conceptual level, our findings raise several questions. In particular, do cerebellar changes co-occur with cortical changes in the ALS-bvFTD syndromes or are they knock-on effects of the substantial cortical changes. Longitudinal monitoring of cortical and cerebellar atrophy would answer this question. Similarly, the role of the cerebellum in the generation of symptoms is not clear. Specifically, how do cerebellar changes modulate, influence or initiate cognitive changes classically seen as ‘cortical’. On an anatomical level there is no doubt that there exists strong cortico-cerebellar circuits involved in cognitive and motor operations [20], [21], [56]. The dense and diverse reciprocal cortico-cerebellar connections, along with the closed-loop architecture of these connections, have implicated the cerebellum in diverse cortical functions suggesting that the cerebellum serves as a major site for cognitive and motor processing. Cognitive, neuropsychiatric and motor disturbances have been reported in humans with focal lesions present only in the cerebellum [57], [70]–[72] and the intensity and nature of these disturbances differs with lesion site and severity. Together, these findings suggest that the cerebellum is involved in more than a modulatory role and that damage to this region impacts on the contralateral cortices. This information will be important to define further in conditions which have both cerebral and cerebellar changes from an early disease stage onwards, such as the FTD and ALS syndromes. It should be noted that to date such cerebellar changes have largely been ignored in these syndromes.

Clinically, these findings highlight the importance of including cerebellar assessment in ALS and bvFTD, both from a diagnostic and staging perspective. Our results suggest that the cerebellum may play an important role in the genesis of symptoms in the ALS-bvFTD syndromes, and that the inclusion of this region in future analyses will advance current understanding of clinical symptoms, particularly since cases with severe cerebellar atrophy in the absence of significant cortical changes have already been reported [26], [55], [65]. Cortical grey matter changes are variably observed in ALS [55] and correlate more with cognitive deficits [73]. Our finding of consistent cerebellar involvement across the ALS-bvFTD continuum together with the unique patterns of atrophy found with neuropsychiatric and motor characteristics suggests that this region may well prove helpful in diagnosing and informing disease progression.

## Supporting Information

Table S1 **ACE-R subscores as a percentage of control values in the different patient groups.** Data are presented as mean ± standard deviation. Table demonstrating that ALS patients performed significantly better on all ACE subscores in comparison to bvFTD and ALS-bvFTD patients. ^a^ ALS compared to bvFTD; ^b^ ALS compared to ALS-bvFTD; ^c^ bvFTD compared to ALS-bvFTD.
(DOCX)
