# Supplementary material for: Cerebellar Integrity in the Amyotrophic Lateral Sclerosis - Frontotemporal Dementia Continuum
Source: PLoS One. 2014 Aug 21;9(8):e105632. doi: 10.1371/journal.pone.0105632 (PMC4140802; doi:10.1371/journal.pone.0105632)
Supplement: Table S1 — ACE-R subscores as a percentage of control values in the different patient groups. Data are presented as mean ± standard deviation. Table demonstrating that ALS patients performed significantly better on all ACE subscores in comparison to bvFTD and ALS-bvFTD patients. a ALS compared to bvFTD; b ALS compared to ALS-bvFTD; c bvFTD compared to ALS-bvFTD. (DOCX) [file pone.0105632.s001.docx]

**Supplementary data**

|  |  |  |  |  |
| --- | --- | --- | --- | --- |
|  | **ALS (n=23)** | **ALS-bvFTD (n=16)** | **bvFTD (n= 23)** | **Significance** |
| **Attention** | 98 ± 4 | 81 ± 23 | 83 ± 22 | p<0.005 ^a, b^ |
| **Memory** | 89 ± 13 | 56 ± 27 | 71 ± 20 | p<0.005 ^a, b^ |
| **Fluency** | 72 ± 26 | 21 ± 27 | 37 ± 25 | p<0.005 ^a, b^ |
| **Language** | 90 ± 11 | 63 ± 22 | 82 ± 17 | p<0.005 ^b, c^ |
| **Visuospatial** | 90 ± 16 | 80 ± 17 | 86 ± 15 | p<0.05 ^b^ |
| **Total** | 89 ± 10 | 62 ± 19 | 74 ± 16 | p<0.005 ^a, b^; p<0.05 ^c^ |

**Table S1. ACE-R subscores as a percentage of control values in the different patient groups.** Data are presented as mean ± standard deviation. ALS patients performed significantly better on all ACE subscores in comparison to bvFTD and ALS-bvFTD patients. ^a^ ALS compared to bvFTD; ^b^ ALS compared to ALS-bvFTD; ^c^ bvFTD compared to ALS-bvFTD.
